# Oroxylin A Directly Targets SRC to Inhibit the PI3K/AKT Signaling Axis in Pancreatic Cancer: An Integrated Bioinformatics and Experimental Study

**DOI:** 10.3390/biom16050685

**Published:** 2026-05-05

**Authors:** Jiajun Zhang, Mengke Song, Huijuan Liu, Yixuan Zhang, Yin Zhang, Zujiao Guan, Song Zhang, Jialun Wang, Shu Zhang, Ying Lv

**Affiliations:** 1 Department of Gastroenterology, Nanjing Drum Tower Hospital Clinical College of Nanjing University of Chinese Medicine, Nanjing 210008, China; 2 Department of Gastroenterology, Nanjing Drum Tower Hospital, The Affiliated Hospital of Nanjing University Medical School, Nanjing 210008, Chinawangjlgastro@163.com (J.W.); zhangsgastro@nju.edu.cn (S.Z.)

**Keywords:** Oroxylin A, pancreatic cancer, flavonoid, apoptosis, SRC/PI3K/AKT, network pharmacology

## Abstract

Oroxylin A (OA), a natural flavonoid, has demonstrated anticancer potential; nevertheless, its precise molecular targets and mechanisms in pancreatic ductal adenocarcinoma (PDAC) remain unclear. To address this gap, we integrated network pharmacology, transcriptomic analysis (GSE101448), and molecular docking, which identified SRC as a core therapeutic target of OA. OA exhibited a strong binding affinity to SRC (−9.0 kcal/mol), and molecular dynamics simulation confirmed stable complex formation (RMSD 0.26 ± 0.02 nm). Combined with functional assays, these findings identify SRC as a critical therapeutic target. Importantly, clinical analysis revealed that SRC was significantly upregulated in PDAC tissues, correlating with poor prognosis. Subsequently, in vitro experiments demonstrated that OA dose-dependently suppressed proliferation, induced G2/M cell cycle arrest and apoptosis, and inhibited migration and invasion in MIA PaCa-2 and PANC-1 cells. These effects were accompanied by downregulation of MMP2 and MMP9. Mechanistically, OA selectively inhibited phosphorylation of SRC (Tyr416), PI3K, and AKT (Ser473) without altering total protein levels, suggesting that the SRC/PI3K/AKT axis is a primary pathway mediating OA’s anticancer activity. Furthermore, in vivo treatment with OA significantly reduced tumor growth in a murine xenograft model without observable toxicity, as indicated by stable body weight, normal organ histology, and unaltered serum liver and kidney function markers. Immunohistochemical analysis further confirmed decreased Ki67 and p-SRC/p-AKT expression, alongside increased cleaved caspase-3, in OA-treated tumors. Collectively, these findings identify SRC as a direct target of OA and demonstrate that OA suppresses PDAC progression through the inhibition of the SRC/PI3K/AKT signaling pathway, thereby supporting its potential as a safe and effective therapeutic candidate for pancreatic cancer.

## 1. Introduction

Pancreatic cancer is among the most fatal malignancies worldwide, characterized by highly aggressive progression and poor clinical outcomes, as reflected by a five-year overall survival rate of approximately 13% [1,2]. Importantly, most patients are diagnosed at an advanced stage due to the absence of early symptoms [3], at which point curative surgical resection is generally not feasible. Furthermore, current chemotherapeutic and radiotherapeutic strategies provide limited clinical benefit and are often associated with substantial toxicity [4]. Collectively, these challenges highlight the urgent need for more effective and well-tolerated therapeutic options.

In recent years, naturally derived bioactive compounds have garnered increasing attention as potential anticancer agents, owing to their diverse pharmacological activities, relatively low toxicity, and favorable safety profiles [5]. Among these compounds, Oroxylin A (OA), a plant-derived flavonoid found in medicinal herbs such as *Scutellaria baicalensis* [6], *Scutellaria lateriflora* [7], and *Aster himalaicus* [8], has demonstrated anti-inflammatory [9], antioxidant [10], antifibrotic [11], and antitumor effects [12]. In addition, epidemiological studies suggest an inverse association between flavonoid consumption and cancer-related mortality [13]. Despite these promising observations, the precise molecular targets of OA in pancreatic cancer, particularly whether it exerts its antitumor effects through direct modulation of oncogenic kinases, remain to be systematically elucidated.

The pathogenesis of pancreatic cancer is closely linked to dysregulated oncogenic signaling networks, among which the PI3K/AKT cascade is frequently hyperactivated, promoting tumor cell survival, proliferation, and metastasis [14,15]. Notably, SRC, a non-receptor tyrosine kinase, functions as a key downstream effector of KRAS signaling and is often overactive in KRAS-mutant pancreatic tumors, where it contributes to therapeutic resistance and disease progression [16,17]. Given that flavonoids have been shown to modulate kinase-driven signaling pathways, we specifically hypothesized that OA exerts its anticancer effects in PDAC by directly binding to and inhibiting the kinase activity of SRC, thereby suppressing the downstream phosphorylation of the PI3K/AKT signaling axis.

To systematically validate this hypothesis, we conducted an integrated evaluation of OA’s anticancer activity using both cellular and animal models of pancreatic cancer. Specifically, a comprehensive approach combining network pharmacology, molecular docking, molecular dynamics simulations, and experimental validation was employed to identify the direct molecular targets of OA and elucidate the underlying mechanisms. Together, our findings clarify how OA suppresses pancreatic cancer progression and provide a strong rationale for its further development as a promising naturally derived therapeutic candidate.

## 2. Materials and Methods

### 2.1. Ethics Approval

All animal experiments were conducted in accordance with the ethical guidelines of the Animal Care and Use Committee at Nanjing Drum Tower Hospital and were approved under protocol number 2024AE01113.

### 2.2. Chemicals and Reagents

OA was obtained from MedChemExpress (Shanghai, China). Cell culture reagents, including DMEM, fetal bovine serum (FBS), and penicillin-streptomycin, were purchased from IMMOCELL (Xiamen, China). Assay kits for CCK-8, BCA protein, cell cycle, Annexin V-EGFP/PI apoptosis, and EdU proliferation were obtained from Abbkine (Wuhan, China), keyGEN (Nanjing, China), Yeasen (Shanghai, China), and Beyotime Biotechnology (Shanghai, China). Cell culture chambers and Matrigel were purchased from Corning (New York, NY, USA) and Mogengel Biotech (Xiamen, China). DMSO was obtained from Macklin (Shanghai, China), and stock OA solutions were prepared in DMSO at final concentrations <0.1%, with DMSO-treated cells serving as vehicle controls. Detection kits for AST, ALT, creatinine (CR), and BUN were obtained from Rayto Biotech (Shenzhen, China). Primary antibodies against PI3K, AKT, SRC, phospho-PI3K, phospho-AKT (Ser473), phospho-SRC (Tyr416), β-actin, Bcl-2, BAX, cleaved caspase-3, cleaved PARP, Ki67, MMP2, and MMP9, as well as HRP-conjugated secondary antibodies, were purchased from Cell Signaling Technology (Danvers, MA, USA), Selleck (Houston, TX, USA), Servicebio (Wuhan, China), Immunoway Biotechnology (San Jose, CA, USA), and Proteintech Group (Wuhan, China).

### 2.3. Computational Analysis

#### 2.3.1. Systematic Bioinformatics Analysis

Gene expression data for GSE101448 were obtained from GEO. Differentially expressed genes (DEGs) were identified using the limma package with thresholds of |log_2_FC| > 1 and an adjusted *p* value (Benjamini–Hochberg FDR) <0.05. These criteria balance the detection of biologically meaningful changes with sufficient statistical power for downstream enrichment analyses and are consistent with standard practices in transcriptomic studies of heterogeneous solid tumors. Concurrently, gene modules associated with PDAC were screened via WGCNA [18,19]. To achieve scale-free topology, an appropriate soft-threshold (β) was selected based on evaluations of scale independence and mean connectivity. The resulting network was then used to identify PDAC-relevant modules.

#### 2.3.2. Network Pharmacology Analysis

Candidate targets of OA were identified from multiple databases, including PharmMapper, SwissTargetPrediction, TargetNet, and the Comparative Toxicogenomics Database (CTD). This multi-database strategy was employed to mitigate the inherent false-negative and false-positive biases of any single prediction algorithm and to capture a comprehensive spectrum of potential OA interactors. Genes associated with pancreatic cancer were obtained from the GeneCards database [20]. The intersection of OA- and PDAC-related targets was determined using the Venny 2.1 online tool. An interaction network for the intersecting targets was constructed via STRING (version 12.0). Hub genes were extracted through MCODE and CytoHubba analyses in Cytoscape (version 3.9.0). Functional enrichment analyses, including GO and KEGG, were performed for the core targets using DAVID (version 6.8), and the results were visualized on an online bioinformatics platform [21].

#### 2.3.3. Molecular Docking

Crystallographic structures of SRC (PDB ID: 1Y57), HIF1A (PDB ID: 1GKC), TIMP2 (PDB ID: 1BR9), and PTGS2 (PDB ID: 5F19) were retrieved from the RCSB Protein Data Bank. The three-dimensional structure of OA was obtained from the PubChem database (CID: 5320315). Protein and ligand structures were prepared using standard procedures, including the addition of non-polar hydrogens, assignment of Gasteiger charges, and conversion to PDBQT format in AutoDock Tools. Molecular docking was subsequently performed with AutoDock Vina (version 1.2.7) to predict the binding affinity of OA for the selected target proteins [22]. To ensure adequate sampling of the binding pockets, grid boxes were centered at predefined coordinates and assigned a uniform size of 26 Å × 26 Å × 26 Å with an exhaustiveness value of 8. The grid centers were as follows: SRC (center_x = 5, center_y = −3, center_z = −4), PTGS2 (center_x = 14, center_y = 49, center_z = 66), HIF1A (center_x = 19, center_y = −17, center_z = −35), and TIMP2 (center_x = 25, center_y = 20, center_z = 16). The docking protocol was validated by redocking the co-crystallized ligand into the SRC binding pocket. The top-ranked pose closely reproduced the native crystal conformation, with a root-mean-square deviation (RMSD) of 1.64 Å, confirming the reliability of the docking settings. Docked conformations were visualized in PyMOL (version 2.6), and complexes with docking scores below −5.0 kcal/mol were considered stable.

#### 2.3.4. Molecular Dynamics Simulations

All molecular dynamics simulations were implemented in the GROMACS (version 2022.3). The SRC-OA complex was prepared using AmberTools22, and GAFF served for ligand parameterization. Hydrogen atoms were introduced and RESP charges were calculated using Gaussian 16W at the B3LYP/6-31G(d) level of theory. Structural optimization was first executed using the steepest descent algorithm, after which the system was subjected to equilibration in NVT/NPT ensembles for 100,000 steps each (coupling constant: 0.1 ps), giving a total of 100 ps of equilibration. The production (molecular dynamics) MD simulation was extended to 100 ns using a 2 fs integration step. The dynamic behavior and stability of the complex were characterized through analysis of RMSD, Rg, RMSF, and MM/GBSA-calculated binding free energies [23].

### 2.4. Cell Culture and Viability Assay

PANC-1 and MIA PaCa-2 cells were obtained from IMMOCELL (Xiamen, China) and cultured in DMEM supplemented with 10% FBS and 1% penicillin-streptomycin. Cells were maintained at 37 °C in a humidified atmosphere containing 5% CO_2_, and the medium was replaced every 2–3 days. For viability assessment, cells were seeded into 96-well plates at a density of 5000 cells per well and allowed to adhere overnight. The cells were then treated with the indicated concentrations of OA for 24 or 48 h. After treatment, 10 μL of CCK-8 reagent was added to each well, and the plates were incubated at 37 °C in the dark. Finally, absorbance at 450 nm was measured using a microplate reader (Tecan, Männedorf, Switzerland) [24].

### 2.5. Clone Formation Assay

PANC-1 and MIA PaCa-2 cells were seeded in 6-well plates at a density of 1000 cells per well and incubated for 24 h to allow adherence. The cultures were then treated with DMSO or OA for 48 h, followed by an additional 14-day incubation [21]. Afterward, colonies were fixed with 4% paraformaldehyde and stained with 0.1% crystal violet. Images were captured using a Nikon microscope (Tokyo, Japan), and colony formation was quantified with ImageJ (version 1.54g).

### 2.6. EdU Staining Assay

PANC-1 and MIA PaCa-2 cells were seeded in 24-well plates at a density of 2 × 10^3^ cells per well and incubated for 24 h. The cells were then treated with DMSO or various concentrations of OA for 48 h. Subsequently, EdU was added at a final concentration of 10 μM and incubated for 2 h [25]. After fixation with 4% paraformaldehyde, cells were permeabilized with 0.3% Triton X-100 for 15 min at room temperature. Fluorescence images were captured using a THUNDER Imager (version 3.9.0, Leica, Wetzlar, Germany) and quantified with ImageJ.

### 2.7. Scratch Assay

Cells (PANC-1 and MIA PaCa-2) were cultured in 6-well plates until reaching approximately 90% confluency. A straight scratch was then made by gently scraping the confluent cell layer with a sterile 200 μL pipette tip to initiate the wound-healing assay [26]. Following the scratch, cells were treated with DMSO or increasing concentrations of OA and incubated for 48 h. Wound images were captured at 0 and 48 h using a Nikon microscope (Tokyo, Japan), and the migration rate was quantified with ImageJ to assess cell motility.

### 2.8. Transwell Migration and Invasion Assays

For the migration assay, cells were seeded into the upper Transwell inserts at densities of 1 × 10^5^ (MIA PaCa-2) and 5 × 10^4^ (PANC-1), while the lower chambers contained 20% FBS as a chemoattractant [27]. The cells were then treated with DMSO or increasing concentrations of OA for 48 h. Migrated cells on the lower surface of the membrane were fixed with 4% paraformaldehyde, stained with 0.1% crystal violet, imaged using a Leica microscope (Leica, Wetzlar, Germany), and quantified with ImageJ.

For the invasion assay, the upper Transwell inserts were precoated with Matrigel (1:8 in serum-free medium, 60 μL per insert) and incubated for 3 h at 37 °C to allow matrix solidification. Cells were subsequently seeded into the Matrigel-coated inserts at the same densities (MIA PaCa-2: 1 × 10^5^; PANC-1: 5 × 10^4^), with 20% FBS-containing medium in the lower chambers as a chemoattractant [28]. After treatment with DMSO or increasing concentrations of OA for 48 h, invaded cells on the lower surface of the membrane were fixed with 4% paraformaldehyde, stained with 0.1% crystal violet, imaged using a Leica microscope (Leica, Wetzlar, Germany), and quantified with ImageJ.

### 2.9. Flow Cytometry

MIA PaCa-2 and PANC-1 cells were seeded in 6-well plates and cultured for 24 h. They were then treated with DMSO or various concentrations of OA for 48 h. Apoptotic cells were quantified using Annexin V-FITC/PI staining. Briefly, 1 × 10^6^ treated cells were collected, centrifuged, and resuspended in binding buffer, then incubated with 5 μL Annexin V-FITC and 10 μL PI for 10 min at room temperature before flow cytometric analysis [29]. For cell cycle analysis, 1 × 10^5^ treated cells were fixed in 70% ethanol at −20 °C overnight. The cells were then incubated with RNase A/PI staining buffer for 40 min at room temperature [30]. Flow cytometry data were acquired and analyzed using FlowJo (version 10.8.1).

### 2.10. Western Blotting

Protein concentration was determined using the BCA assay. Protein samples were separated by SDS-PAGE and subsequently transferred onto PVDF membranes. After blocking, membranes were incubated first with specific primary antibodies and then with the corresponding secondary antibodies. Immunoreactive signals were detected using ECL and quantified with ImageJ [24]. Western blot original images can be found in Supplementary Materials.

### 2.11. Subcutaneous Xenograft Tumor Model and In Vivo Safety Assessment

Male BALB/c nude mice (5 weeks old, 17–19 g; *n* = 10) were obtained from GemPharmatech (Nanjing, China) and maintained under SPF conditions at the Experimental Animal Center of Nanjing Drum Tower Hospital, with controlled temperature (22 ± 3 °C), 50% humidity, and a 12 h light/dark cycle. To establish the xenograft model [31], 1 × 10^7^ PANC-1 cells suspended in saline were injected subcutaneously into the right thigh.

Mice were randomly assigned to a saline control or OA treatment group (300 mg/kg, oral gavage; *n* = 5 per group). OA was suspended in saline containing 0.5% CMC-Na and 0.5% Tween-80 and administered via oral gavage at a dose of 300 mg/kg every two days for 21 days. Tumor volume and body weight were recorded every three days throughout the study. At the end of the experiment, tumors and major organs were collected for histological analysis. All procedures complied with the 3Rs principles and institutional guidelines for animal welfare. Serum samples were collected to assess in vivo safety. Hepatic and renal function were evaluated by measuring AST, ALT, CR, and BUN levels using commercial assay kits and a fully automated biochemical analyzer (Rayto, Shenzhen, China).

### 2.12. Histological and Immunohistochemical Analysis

The liver, kidney, heart, lung, spleen, and tumor tissues were collected for histopathological analysis. Tissue sections were prepared through routine histological procedures, including fixation, paraffin embedding, sectioning, and H&E staining [32]. The stained sections were then digitized using a high-resolution slide scanner (Leica, Wetzlar, Germany) and analyzed with SlideViewer software (version 2.7.0). Immunohistochemistry was performed to detect Ki67, cleaved caspase-3, p-SRC (Tyr416), p-PI3K, p-AKT (Ser473), MMP9, and MMP2 in mouse tumor tissues. Tumor sections were incubated with specific primary antibodies, followed by the corresponding secondary antibodies. Signals were visualized using freshly prepared diaminobenzidine, and the sections were counterstained with hematoxylin [33].

### 2.13. Statistical Analysis

All statistical analyses were performed using GraphPad Prism (version 9.0) and are presented as means ± SD. Comparisons between two groups were conducted using a two-tailed unpaired Student’s t-test, while one-way ANOVA was applied for analyses involving more than two groups. IC_50_ values were determined via nonlinear regression. A *p* value of less than 0.05 was considered statistically significant.

## 3. Results

### 3.1. OA Triggers Apoptosis in Pancreatic Carcinoma Cells

**Figure 1:**
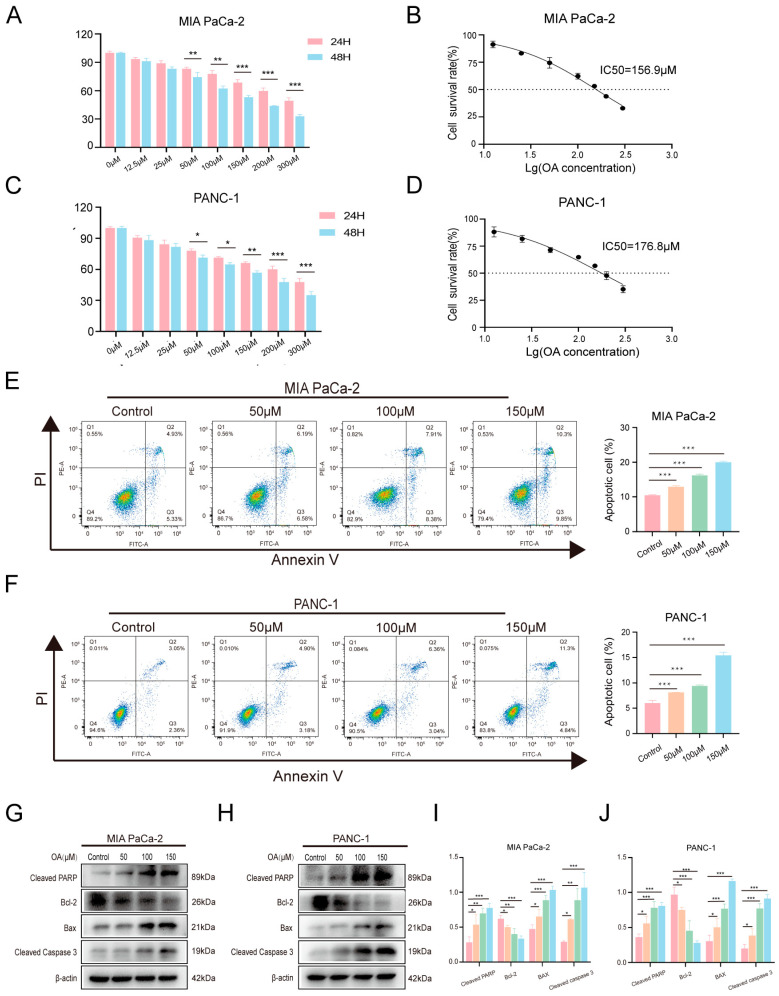
OA induces apoptosis in PDAC cells. (**A**) Effects of OA (0–300 μM) on the viability of MIA PaCa-2 cells after 24 and 48 h of treatment. (**B**) IC_50_ values of OA in MIA PaCa-2 cells at 48 h. (**C**) Effects of OA (0–300 μM) on the viability of PANC-1 cells after 24 and 48 h of treatment. (**D**) IC_50_ values of OA in PANC-1 cells at 48 h. (**E**) Flow cytometric analysis of apoptosis in MIA PaCa-2 cells following OA treatment. (**F**) Flow cytometric analysis of apoptosis in PANC-1 cells following OA treatment. (**G**,**H**) Western blot analysis of cleaved PARP, BCL-2, BAX, and cleaved caspase-3 in MIA PaCa-2 and PANC-1 cells after OA treatment. (**I**,**J**) Quantitative analysis of protein expression in MIA PaCa-2 and PANC-1 cells. Data are presented as the mean ± SD from independent experiments performed in biological duplicates. * *p* < 0.05, ** *p* < 0.01, *** *p* < 0.001 versus controls.

OA-induced cytotoxicity was evaluated using the CCK-8 assay. OA significantly reduced the viability of MIA PaCa-2 and PANC-1 cells in a dose-dependent manner (Figure 1A,C). The 48-h IC_50_ values were 156.9 μM for MIA PaCa-2 cells (Figure 1B) and 176.8 μM for PANC-1 cells (Figure 1D). To assess the selectivity of OA, its effects were also examined in normal human pancreatic ductal epithelial cells (hTERT-HPNE). As shown in Supplementary Figure S1A,B, OA exhibited markedly weaker cytotoxicity in hTERT-HPNE cells, with a 48-h IC_50_ value of 271.4 μM. This result indicates that OA preferentially targets pancreatic cancer cells. Flow cytometric analysis confirmed that OA induced apoptosis after 48 h of treatment, and the proportion of apoptotic cells increased with OA concentration (Figure 1E,F). Western blot analysis further showed that increasing OA concentrations elevated the expression of BAX, cleaved caspase-3, and cleaved PARP, while progressively reducing BCL-2 expression (Figure 1G–J). In addition, OA treatment dose-dependently increased cleaved caspase-9 levels in both cell lines (Supplementary Figure S2), further confirming activation of the intrinsic apoptotic pathway.

### 3.2. OA Impairs Pancreatic Cancer Cell Growth and Causes G2/M Phase Arrest

**Figure 2:**
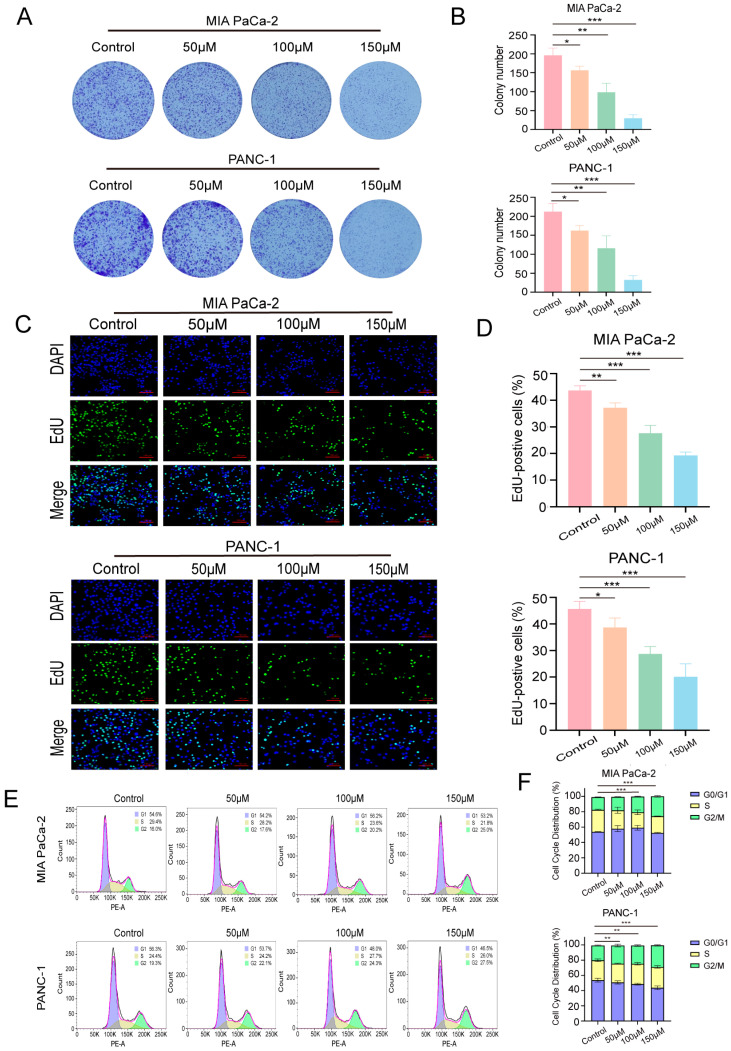
OA inhibits proliferation in PDAC cells. (**A**,**B**) Colony formation of MIA PaCa-2 and PANC-1 cells after 14 days of OA treatment. (**C**,**D**) EDU staining of cells following 48 h of OA treatment (magnification: 20×). (**E**,**F**) Flow cytometry analysis of cell cycle distribution in MIA PaCa-2 and PANC-1 cells, showing the percentages of cells in G0/G1, S, and G2/M phases. Data are expressed as means ± SD from independent experiments performed in biological duplicates. * *p* < 0.05, ** *p* < 0.01, *** *p* < 0.001 versus controls.

Colony-forming assays demonstrated that OA markedly suppressed the clonogenic growth of cells in a dose-dependent manner (Figure 2A,B). In line with these findings, EdU incorporation assays revealed a significant reduction in cellular proliferative capacity following OA treatment (Figure 2C,D). Moreover, cell cycle analysis showed an accumulation of cells in the G2/M phase after OA exposure (Figure 2E,F). Taken together, these results indicate that OA substantially impairs pancreatic cancer cell proliferation in vitro.

### 3.3. OA Inhibits Migration and Invasion of Pancreatic Cancer Cells

**Figure 3:**
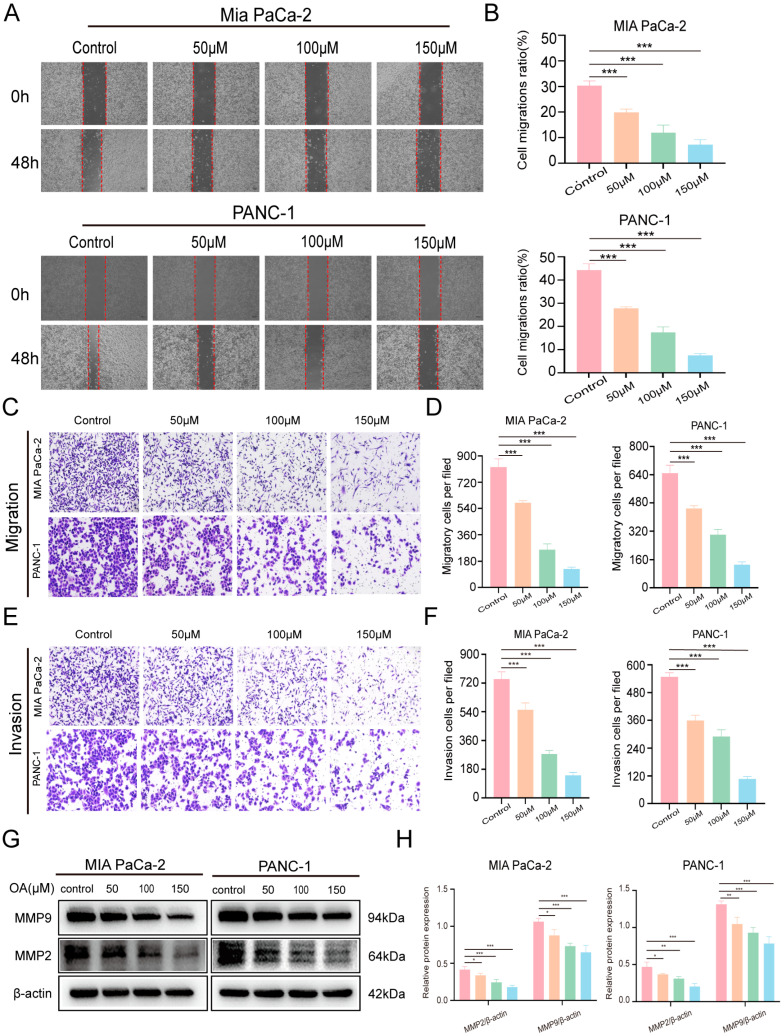
OA inhibits the motility and invasive potential of PDAC cells. (**A**,**B**) Scratch assay showing the migratory capacity of MIA PaCa-2 and PANC-1 cells after 48 h of OA treatment (magnification: 4×). (**C**,**D**) Transwell migration assay assessing the migration of pancreatic carcinoma cells after 48 h of OA treatment (magnification: 10×). (**E**,**F**) Transwell invasion assay evaluating the invasive ability of pancreatic tumor cells after 48 h of OA treatment (magnification: 10×). (**G**,**H**) Western blot analysis of MMP2 and MMP9 expression. Data are presented as means ± SD from independent experiments with biological duplicates. * *p* < 0.05, ** *p* < 0.01, *** *p* < 0.001 versus controls.

OA treatment for 48 h markedly suppressed the migration of MIA PaCa-2 and PANC-1 cells compared with controls, with the inhibitory effect increasing in a dose-dependent manner (Figure 3A,B). Consistently, Transwell migration assays confirmed a significant decrease in migrated cells after OA treatment (Figure 3C,D). Likewise, Transwell invasion assays showed that OA substantially impaired the invasive capacity of these cells in a dose-dependent manner (Figure 3E,F). Western blot analysis further revealed that higher OA concentrations reduced MMP2 and MMP9 expression in both cell lines (Figure 3G,H). Overall, these results demonstrate that OA exerts strong anti-migratory and anti-invasive effects on PDAC cells in vitro.

### 3.4. Network Pharmacology Identifies Potential Molecular Targets of OA in Pancreatic Cancer

**Figure 4:**
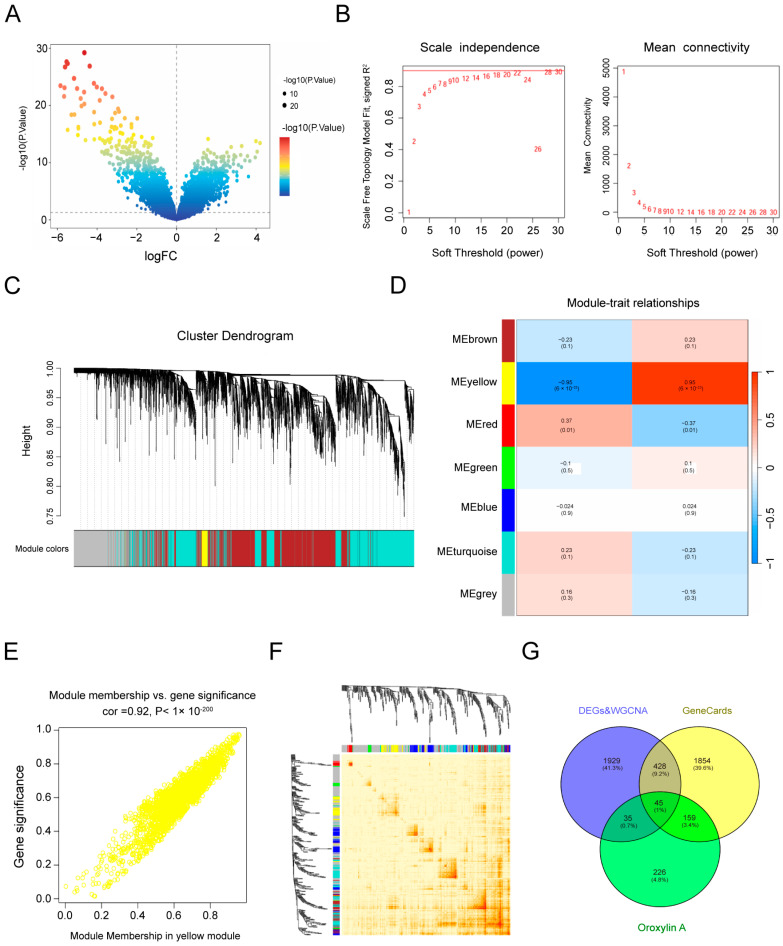
Identification of pancreatic cancer-related differentially expressed genes by integrated bioinformatics and network pharmacology analysis. (**A**) Volcano plots of DEGs. (**B**) Scale-free topology model fit (R^2^) versus soft-thresholding power, showing an R^2^ of 0.9 at β = 14. (**C**) Cluster trend analysis. The upper panel displays a gene-level clustering dendrogram, while the lower panel shows gene modules. (**D**) Heatmap of correlations between control and PDAC-associated modules, with red indicating positive correlations and blue indicating negative correlations. (**E**) Scatter plot of module membership versus gene significance. (**F**) Topological overlap matrix (TOM), where darker colors indicate stronger gene–gene interactions. (**G**) Venn diagram showing the number of intersecting genes among the GEO database, disease databases, and drug databases.

The experiments above confirmed that OA inhibits pancreatic cancer cells; however, its precise molecular targets remain unknown. To investigate these targets, network pharmacology was employed. Using the chemical structure of OA and the term “Oroxylin A” as search queries, 104, 54, 295, and 130 candidate targets were retrieved from the SwissTargetPrediction, CTD, PharmMapper, and TargetNet databases, respectively. After removing duplicates, 465 OA-associated targets were identified. Pancreatic cancer–related genes were then collected from multiple sources. From GeneCards, 5047 genes were retrieved, of which 2486 were retained based on relevance scores. Concurrently, transcriptomic analysis of the GSE101448 dataset revealed 1563 DEGs, comprising 652 upregulated and 911 downregulated genes (Figure 4A).

Furthermore, weighted gene co-expression network analysis (WGCNA) was performed on the GSE101448 dataset to further explore gene interactions. Sample clustering and heatmap analyses were conducted, followed by module identification using the topological overlap matrix (Figure 4B–D). Among the modules, the yellow module showed the strongest correlation with PDAC. Within this module, gene significance correlated positively with module membership (cor = 0.92, *p* < 1 × 10^−200^), defining these genes as core PDAC-related genes (Figure 4E,F). Genes identified from WGCNA were merged with DEGs after removing duplicates, yielding 2437 PDAC-related genes. Finally, overlap analysis integrating OA-associated targets (465 genes), GeneCards-derived pancreatic cancer genes (2486 genes), and GEO-derived PDAC genes (2437 genes) revealed 45 overlapping genes (Figure 4G). These genes were considered potential therapeutic targets of OA in pancreatic cancer.

### 3.5. SRC Is Identified as a Pharmacologically Relevant Target of OA

**Figure 5:**
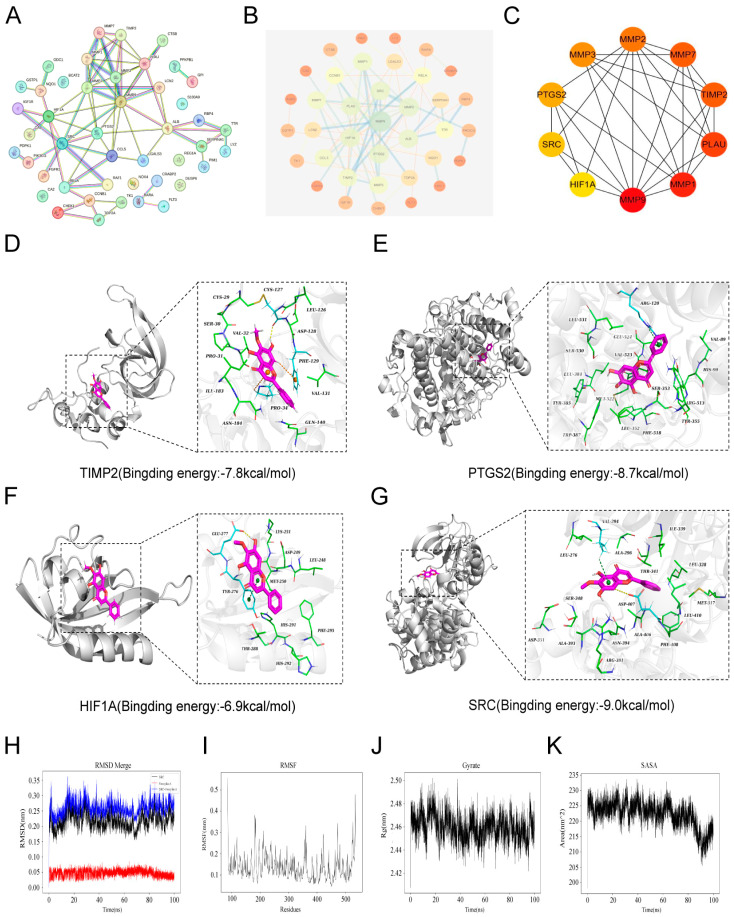
Identification of OA targets in PDAC. (**A**,**B**) PPI network of OA-related targets in pancreatic cancer. (**C**) PPI network of key OA-related targets identified using the CytoHubba algorithm. (**D**–**G**) Docking models showing interactions between OA and TIMP2 (**D**), PTGS2 (**E**), HIF1A (**F**), and SRC (**G**). (**H**) RMSD of OA bound to SRC. (**I**) RMSF of OA bound to SRC. (**J**) Radius of gyration (Rg) of OA bound to SRC. (**K**) Solvent-accessible surface area (SASA) of OA bound to SRC.

STRING analysis generated a PPI network for 45 shared targets at a confidence score ≥0.700. The Cytoscape-rendered network included 37 nodes and 73 edges, representing proteins and their interactions (Figure 5A,B). Hub genes were identified using the CytoHubba algorithm, which ranked the top 10 genes as MMP9, SRC, PTGS2, HIF1A, MMP3, MMP2, MMP7, TIMP2, PLAU, and MMP1 (Figure 5C). Furthermore, two highly connected clusters were identified using the MCODE algorithm. Cluster 1 (Supplementary Figure S3A) contained MMP9, SRC, PTGS2, HIF1A, MMP3, MMP2, MMP7, TIMP2, PLAU, and MMP1, whereas Cluster 2 (Supplementary Figure S3B) consisted of CCNB1, TOP2A, CHEK1, and TK1. Notably, ten genes were shared between the CytoHubba and MCODE results, suggesting that these genes may represent key potential targets of OA in pancreatic cancer.

To identify core candidate targets of OA, molecular docking was performed to evaluate its binding affinity toward hub proteins. Targets with binding energies ≤−5.0 kcal/mol were retained for further analysis. The AutoDock Vina results indicated that TIMP2 (Figure 5D), PTGS2 (Figure 5E), HIF1A (Figure 5F), and SRC (Figure 5G) all exhibited binding energies below the threshold. Among these targets, SRC exhibited a favorable docking energy of −9.0 kcal/mol with OA. Although this predicted interaction requires further validation, SRC was prioritized for subsequent investigation because of its role as an upstream regulator of the PI3K/AKT signaling pathway identified in KEGG enrichment analysis and its established relevance as a therapeutic target in KRAS-driven pancreatic cancer. The lowest-energy OA-SRC complex was therefore subjected to molecular dynamics simulation to further evaluate the stability of the predicted binding interaction.

MD simulation analysis demonstrated that the RMSD of the OA-SRC complex remained stable at 0.26 ± 0.02 nm throughout the simulation (Figure 5H), indicating structural stability. RMSF analysis showed an average residue fluctuation of 0.14 ± 0.06 nm (Figure 5I), suggesting limited flexibility of SRC residues upon OA binding. Additionally, the radius of gyration (Rg) of the complex was maintained at 2.46 ± 0.01 nm (Figure 5J), reflecting a stable overall conformation. The solvent-accessible surface area (SASA) remained at 222.34 ± 4.28 nm^2^ (Figure 5K), indicating preserved structural integrity under simulated physiological conditions. Furthermore, MM/GBSA analysis of the equilibrated MD trajectory showed that the total binding free energy (ΔG_bind) of the SRC-OA complex was −39.52 ± 2.42 kcal/mol. This favorable binding was primarily driven by van der Waals (ΔE_vdW = −40.52 ± 1.52 kcal/mol) and electrostatic (ΔE_elec = −21.03 ± 0.67 kcal/mol) interactions (Supplementary Table S1).

### 3.6. Elevated SRC Expression Predicts Poor Prognosis in Pancreatic Cancer

**Figure 6:**
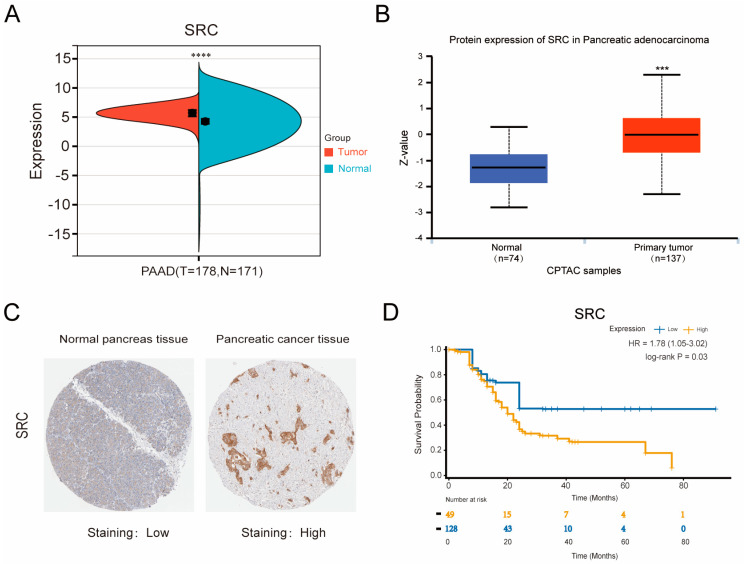
Clinical significance of SRC in PDAC. (**A**) SRC mRNA levels in PDAC and normal pancreatic tissue (SangerBox). (**B**) SRC protein levels in PDAC and adjacent non-tumor tissue (UALCAN). (**C**) SRC protein expression in normal pancreas and PDAC as evaluated by immunohistochemistry (HPA). (**D**) Association between SRC mRNA expression and overall survival of PDAC patients in the TCGA database. *** *p* < 0.001, **** *p* < 0.0001 versus controls.

Analyses of public databases were conducted to evaluate the clinical significance of SRC in pancreatic cancer. Transcriptomic data from the SangerBox platform, integrating TCGA and GTEx datasets, showed that SRC mRNA was markedly upregulated in pancreatic adenocarcinoma compared with normal pancreatic tissues (Figure 6A). Similarly, UALCAN proteomic analysis (CPTAC/ICPC) revealed elevated SRC protein levels in tumor tissues (Figure 6B), and immunohistochemistry data from the HPA database further confirmed higher SRC expression in pancreatic cancer relative to normal tissues (Figure 6C). Together, these multi-database analyses demonstrate that SRC is consistently overexpressed at both the transcriptional and protein levels in pancreatic cancer. Moreover, survival analysis indicated that high SRC expression correlates with significantly poorer overall survival (Figure 6D).

### 3.7. OA Modulates the SRC/PI3K/AKT Signaling in Pancreatic Cancer Cells

**Figure 7:**
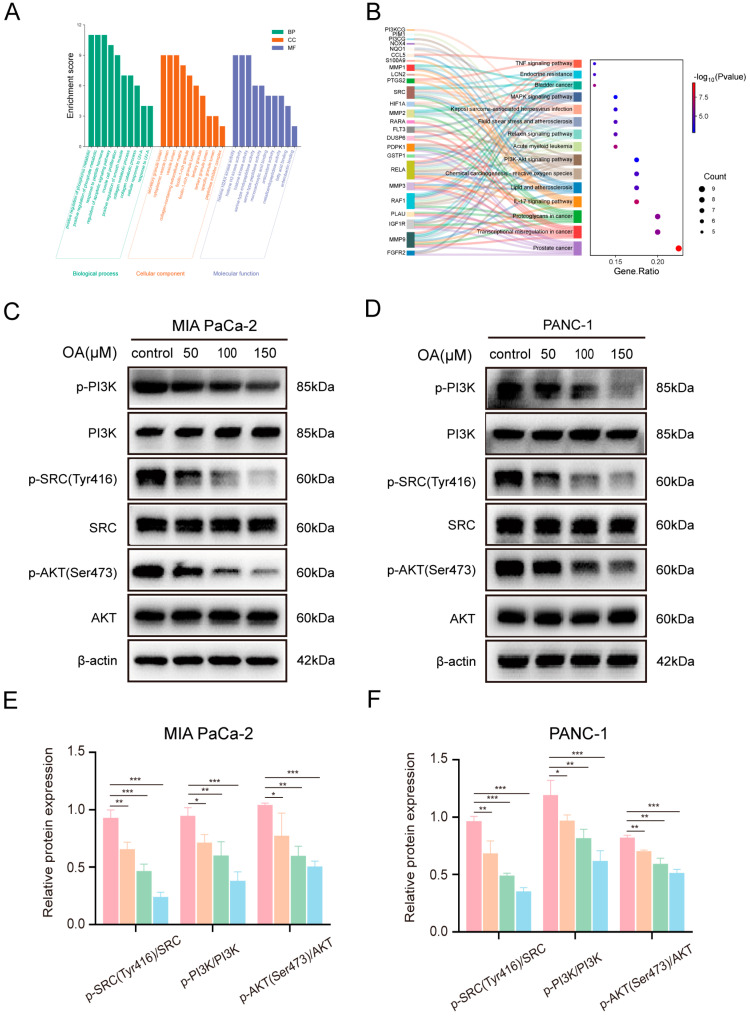
Inhibitory effects of OA on SRC/PI3K/AKT signaling in pancreatic tumor cells. (**A**) GO term enrichment shown as a bar plot. (**B**) KEGG pathway enrichment visualized with a Sankey plot. (**C**) Protein levels of p-SRC (Tyr416), SRC, p-PI3K, PI3K, p-AKT (Ser473), and AKT in OA-treated MIA PaCa-2 cells. (**D**) Protein levels of p-SRC (Tyr416), SRC, p-PI3K, PI3K, p-AKT (Ser473), and AKT in OA-treated PANC-1 cells. (**E**) Quantification of the indicated proteins in MIA PaCa-2 cells. (**F**) Quantification of the indicated proteins in PANC-1 cells. Data are presented as means ± SD from independent experiments with biological duplicates. * *p* < 0.05, ** *p* < 0.01, *** *p* < 0.001 versus controls.

After SRC was identified as a potential OA target, network pharmacology was applied to investigate the biological functions and signaling pathways of OA-related targets. GO enrichment analysis of PPI network-derived targets revealed significant overrepresentation of processes regulating phosphorus and phosphate, apoptosis, and collagen catabolism (Figure 7A). Enriched molecular functions included histone kinase, metalloendopeptidase, and serine-type peptidase activities. KEGG analysis highlighted the PI3K/AKT pathway as a key signaling axis for OA-associated targets (Figure 7B). Given the established regulatory link between SRC and PI3K/AKT signaling, we assessed the effects of OA on this axis in pancreatic cancer cells. Western blotting showed marked reductions in p-SRC (Tyr416), p-PI3K, and p-AKT (Ser473) levels in MIA PaCa-2 and PANC-1 cells following OA treatment (Figure 7C,D). Notably, phosphorylated protein levels decreased in a concentration-dependent manner, while total protein levels remained largely unchanged (Figure 7E,F).

### 3.8. OA Suppresses Pancreatic Tumor Growth In Vivo with Favorable Safety Profile

**Figure 8:**
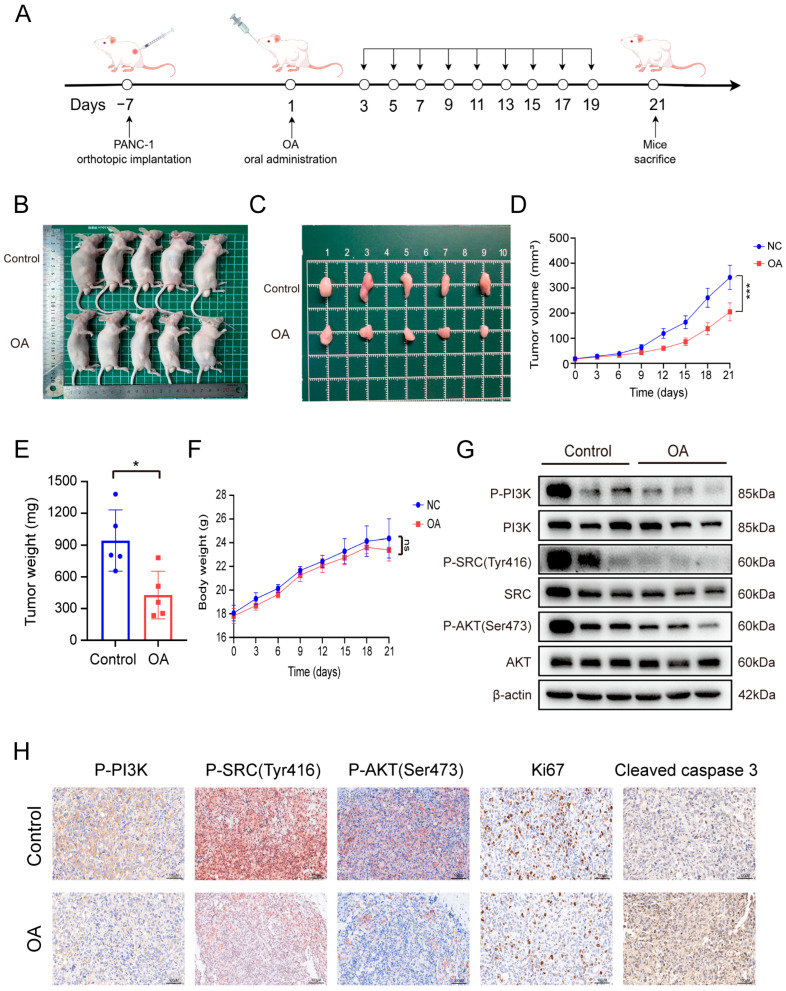
OA suppresses growth of subcutaneous pancreatic tumors via inhibition of SRC/PI3K/AKT signaling. (**A**) Schematic illustration of the subcutaneous PDAC xenograft model and treatment regimen. (**B**) Photographs of mice after humane euthanasia. (**C**) Representative images of PDAC tumors. (**D**) Tumor volume curves of mice over time. (**E**) Average tumor weight. (**F**) Average body weight of mice. (**G**) Western blot analysis of p-PI3K, total PI3K, p-SRC (Tyr416), total SRC, p-AKT (Ser473), and total AKT in tumor tissues. (**H**) Immunohistochemical staining of p-SRC (Tyr416), p-PI3K, p-AKT (Ser473), Ki67, and cleaved caspase-3 in tumor sections. * *p* < 0.05, *** *p* < 0.001 versus controls. Scale bars: 100 μm (H).

**Figure 9:**
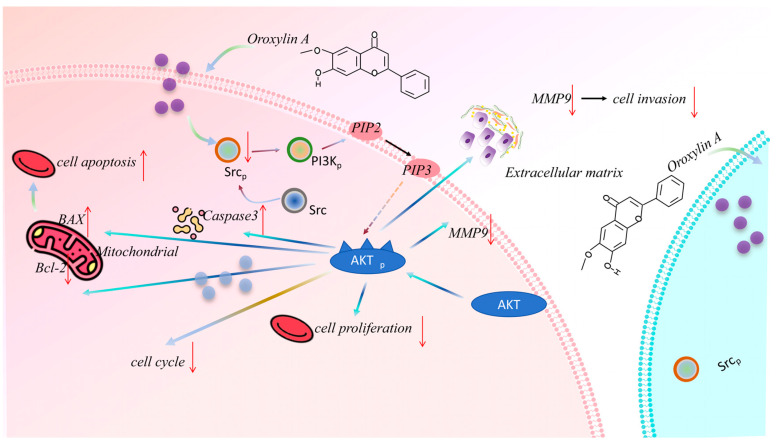
Schematic model of the antitumor mechanism of OA in pancreatic cancer. OA directly targets SRC, thereby inhibiting SRC phosphorylation at Tyr416 and suppressing the downstream PI3K/AKT signaling axis. This suppression induces G2/M cell-cycle arrest and activates cleaved caspase-3, leading to intrinsic apoptosis. OA also inhibits tumor cell migration and invasion through the downregulation of MMP2 and MMP9. Downward arrows (↓) indicate inhibitory effects, and upward arrows (↑) indicate activation or upregulation. The dashed outline highlights the major signaling pathway targeted by OA.

To further assess the therapeutic efficacy of OA in vivo, subcutaneous xenograft models were established by implanting PANC-1 cells into BALB/c nude mice (Figure 8A). OA treatment significantly inhibited tumor growth compared with the untreated group (Figure 8B,C), resulting in marked reductions in both tumor volume and weight (Figure 8D,E). Importantly, OA did not affect body weight (Figure 8F), and H&E staining of major organs revealed no detectable tissue damage (Supplementary Figure S4A). Serum levels of ALT, AST (Supplementary Figure S4B), creatinine (CR), and blood urea nitrogen (BUN) (Supplementary Figure S4C) remained within normal ranges, further supporting the favorable safety profile of OA. Mechanistically, Western blot analysis of tumor tissues showed that OA decreased phosphorylated SRC (Tyr416), PI3K, and AKT (Ser473) levels, consistent with our in vitro results (Figure 8G). Immunohistochemical staining confirmed reduced Ki67 expression and elevated cleaved caspase-3 levels, indicating suppressed proliferation and enhanced apoptosis. Moreover, IHC demonstrated inhibition of SRC/PI3K/AKT phosphorylation in tumor tissues (Figure 8H), and MMP2 and MMP9 expression was also decreased following OA treatment (Supplementary Figure S4D). In summary, these findings indicate that OA suppresses tumor growth in vivo, at least in part, by inhibiting the SRC/PI3K/AKT signaling pathway, as evidenced by the reduced phosphorylation of SRC (Tyr416), PI3K, and AKT (Ser473) in tumor tissues (Figure 9). Furthermore, the upregulation of cleaved caspase-3 confirms the induction of apoptosis downstream of this signaling blockade (Figure 9).

## 4. Discussion

In this study, we demonstrate the pharmacological antitumor potential of OA in pancreatic cancer, with its biological effects closely linked to modulation of SRC-mediated PI3K/AKT signaling. Using both cellular and mouse models, OA consistently attenuated malignant phenotypes of pancreatic tumor cells, including proliferation, migration, and survival. Importantly, OA exhibited a favorable safety profile in mice. Histopathological examination of major organs, together with stable body weight during treatment, indicates that OA does not induce overt systemic toxicity, consistent with previous clinical observations reporting its good tolerability. The exploration of natural compounds as anticancer agents has garnered increasing interest, primarily due to their broad bioactivity and relatively low toxicity. Numerous phytochemicals, including curcumin [34], berberine [35], baicalein [36], and piperine [37], have demonstrated antitumor efficacy across multiple cancer types, such as lung [38], colon [39], and breast cancers [40]. These compounds often exert pleiotropic effects, allowing them to modulate cancer-associated signaling networks while sparing normal tissues [41]. Although OA has been reported to exhibit anticancer activity in liver [42], gastric [43], breast [44], and colon cancers [45], its role in pancreatic cancer remains insufficiently characterized. Therefore, mechanistic studies are warranted to elucidate its effects in this highly aggressive malignancy.

Aberrant SRC activation is a well-recognized hallmark of pancreatic cancer and contributes to tumor growth, survival, and metastatic progression [46]. By stimulating downstream signaling networks, SRC promotes sustained proliferation, resistance to apoptosis, and enhanced migratory capacity. These cellular processes are tightly interconnected, especially under oncogenic stress. For example, in response to DNA damage or metabolic disturbances, cells may undergo cell cycle arrest to facilitate DNA repair. However, persistent damage can trigger apoptotic programs to eliminate compromised cells [47].In this context, OA treatment induced G2/M checkpoint arrest and apoptosis in MIA PaCa-2 and PANC-1 cells. These effects align with previous reports of OA-mediated cell cycle disruption and apoptosis in other cancer models [48,49]. Together, the findings suggest that OA disrupts cell cycle progression, thereby sensitizing pancreatic cancer cells to apoptotic signaling. Metastatic dissemination is a major contributor to poor clinical outcomes in pancreatic cancer, a process facilitated by the tumor’s dense and dynamic extracellular matrix [50]. In the present study, OA significantly reduced cancer cell motility and invasiveness, accompanied by downregulation of matrix metalloproteinases. This inhibitory effect on motility is consistent with observations in other tumor types and supports the notion that OA may interfere with cytoskeletal remodeling and extracellular matrix degradation—both essential for tumor invasion [51,52].

To further elucidate the molecular basis of OA activity, we integrated network pharmacology with molecular docking and molecular dynamics simulations. This combined approach identified SRC as a pharmacologically relevant signaling node that may mediate the antitumor effects of OA. However, these in silico analyses are hypothesis-generating and do not provide definitive evidence of direct binding or target specificity. Although the predicted binding conformation and favorable free energy estimates suggest a plausible interaction, rigorous biophysical assays, such as CETSA, SPR, and MST, are required for formal validation. Importantly, the computational predictions are supported by experimental findings. OA markedly reduced the phosphorylation of SRC (Tyr416), PI3K, and AKT (Ser473) in both cellular and xenograft models, while total protein levels remained unchanged. This selective post-translational modulation is consistent with interference at the level of the SRC kinase domain. Collectively, these results indicate that inhibition of the SRC/PI3K/AKT signaling axis contributes, at least in part, to the antitumor activity of OA.

Although these in silico analyses do not constitute definitive target validation, they provide a mechanistic framework suggesting that OA may interact with SRC to modulate downstream signaling cascades. Consistent with these predictions, KEGG enrichment analysis highlighted the PI3K/AKT pathway as a major signaling cascade associated with OA. Experimental validation confirmed that OA markedly suppressed phosphorylation of SRC, PI3K, and AKT in both cellular and xenograft models. Collectively, these findings indicate that inhibition of the SRC/PI3K/AKT cascade contributes, at least in part, to OA’s observed antitumor effects. Notably, the in vitro IC_50_ values of OA (156.9–176.8 μM) are relatively high, reflecting the moderate intrinsic potency characteristic of multi-target flavonoids [53,54]. Nevertheless, OA demonstrates significant antitumor efficacy in vivo at a well-tolerated dose (300 mg/kg). This efficacy, together with a broad safety window evidenced by unchanged organ function and stable serum biomarkers, supports the notion that such micromolar concentrations are pharmacologically attainable and therapeutically relevant. From a therapeutic perspective, managing pancreatic cancer remains challenging due to the limited efficacy and substantial toxicity of standard chemotherapy [55]. Agents that modulate oncogenic signaling while maintaining favorable safety profiles are therefore of considerable interest. Given that PI3K/AKT signaling is a well-established therapeutic target in pancreatic cancer [14], OA’s ability to attenuate SRC-mediated PI3K/AKT activation suggests potential utility either as a standalone agent or in combination regimens. This notion aligns with previous findings demonstrating cooperative interactions between flavonoids and other anticancer agents [56].

Clinical relevance is supported by a phase I trial showing that oral OA administration up to 2400 mg is safe and well tolerated in healthy volunteers [57]. Additionally, multiple preclinical studies have demonstrated that OA suppresses tumor growth in vivo across a range of doses [12,44,45]. Given the multi-target and multi-pathway nature of flavonoids [58,59,60,61], OA may be particularly suitable for combination strategies designed to enhance therapeutic efficacy and overcome drug resistance [62]. From a translational perspective, its low toxicity further supports evaluation in combination with standard chemotherapeutics such as gemcitabine or 5-FU. Hyperactivation of the SRC/PI3K/AKT signaling axis contributes to gemcitabine resistance; thus, OA-mediated inhibition of this pathway may enhance chemosensitivity [63]. Consistent with this rationale, previous studies have shown that flavonoids or SRC inhibitors can synergize with gemcitabine in preclinical PDAC models [64,65]. Therefore, evaluating OA in combination with gemcitabine or 5-FU represents a logical next step toward clinical translation. Nonetheless, several limitations should be acknowledged. First, this study relied on established cell lines and a subcutaneous xenograft model, which may not fully capture the heterogeneity and stromal complexity of pancreatic tumors. Second, although the relatively small sample size (*n* = 5 per group) is sufficient to demonstrate primary tumor growth inhibition based on prior studies and power calculations, it may limit the generalizability of safety and pharmacodynamic findings. Therefore, future investigations using patient-derived xenograft and genetically engineered mouse models are warranted to further validate the translational relevance of these findings. Moreover, although the SRC/PI3K/AKT signaling axis was identified as a key pathway mediating OA activity, the involvement of additional molecular targets cannot be excluded. Finally, comprehensive pharmacokinetic analyses and long-term toxicity studies in relevant disease models will be essential before clinical application.

## 5. Conclusions

Taken together, this study identifies SRC as a direct and pharmacologically relevant target of OA in pancreatic cancer. This conclusion is supported by an integrated strategy combining network pharmacology, molecular docking, molecular dynamics simulations, and multi-dimensional experimental validation. Mechanistically, OA selectively inhibits SRC phosphorylation at Tyr416 and the downstream PI3K/AKT signaling cascade, thereby suppressing tumor cell proliferation, inducing G2/M arrest and apoptosis, and impairing migration and invasion in vitro. The in vivo relevance of this mechanism is confirmed in a xenograft model, where OA effectively suppresses tumor growth while preserving body weight, maintaining normal organ histology, and keeping serum liver and kidney biomarkers within normal ranges. Collectively, these results establish OA as a promising phytotherapeutic candidate for pancreatic cancer and uncover a previously unrecognized mechanism involving the direct targeting of the SRC/PI3K/AKT axis, providing a strong rationale for further preclinical and clinical development.

## Data Availability

The original contributions presented in this study are included in the article/Supplementary Materials. Further inquiries can be directed to the corresponding authors.

## Supplementary Materials

The following supporting information can be downloaded at: https://www.mdpi.com/article/10.3390/biom16050685/s1. Figure S1. Effects of OA on the viability of normal pancreatic epithelial cells (hTERT-HPNE). Figure S2. OA dose-dependently increased cleaved caspase-9 expression in MIA PaCa-2 (A,C) and PANC-1 (B,D) cells after 48 h of treatment. Figure S3. The protein interaction network diagram of key targets in OA-pancreatic cancer in MCODE algorithm. Figure S4. H&E-stained histology images of mouse organs and IHC images. Table S1. MM/GBSA binding free energy components of SRC-Oroxylin A complex. File S1. WB original images.
