# Supplementary material for: Oroxylin A Directly Targets SRC to Inhibit the PI3K/AKT Signaling Axis in Pancreatic Cancer: An Integrated Bioinformatics and Experimental Study
Source: Biomolecules. 2026 May 5;16(5):685. doi: 10.3390/biom16050685 (PMC13204231; doi:10.3390/biom16050685)
Supplement: Supplementary file 1 [file biomolecules-16-00685-s001.zip › biomolecules-4274030-supplementary.pdf]

Supplementary Date

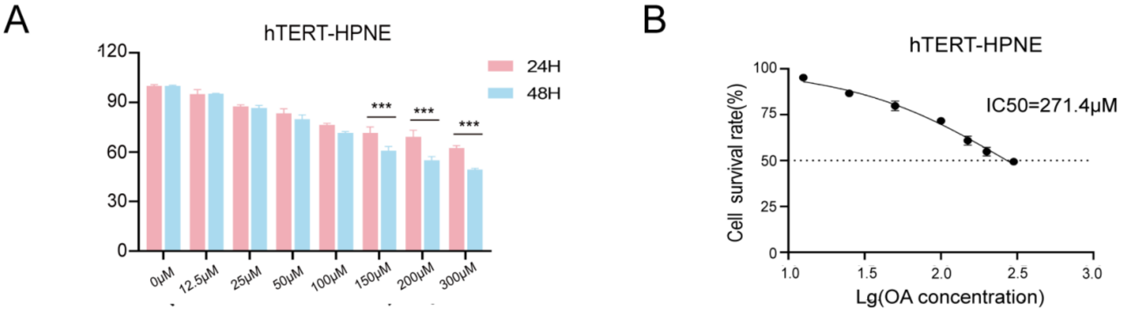

Supplementary Figure S1. Effects of OA on the viability of normal pancreatic epithelial cells (hTERT-HPNE). (A) Cell viability following OA treatment for 24 or 48 h, as determined by the CCK-8 assay. (B) Dose–response curve and corresponding IC_{50} value (271.4 μM) after 48 h of treatment. Data are presented as the mean ± SD from three independent experiments. ****P* < 0.001versus the control group.

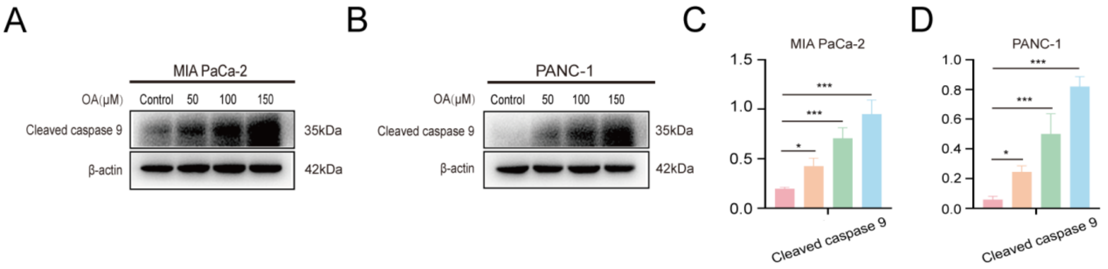

Supplementary Figure S2. OA dose-dependently increased cleaved caspase-9 expression in MIA PaCa-2 (A, C) and PANC-1 (B, D) cells after 48 h of treatment. β-actin was used as the loading control. Quantitative analysis is presented below. Data are expressed as the mean ± SD from three independent experiments. **P* < 0.05, ***P* < 0.01, ****P* < 0.001 versus the control group.

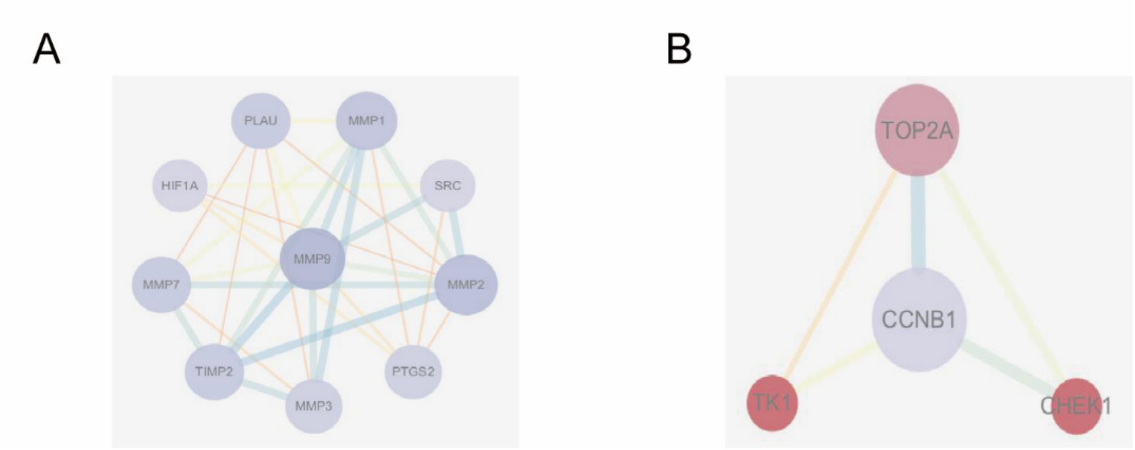

Supplementary Figure S3. The protein interaction network diagram of key targets in OA-pancreatic cancer in MCODE algorithm. (A) Cluster 1. (B) Cluster 2.

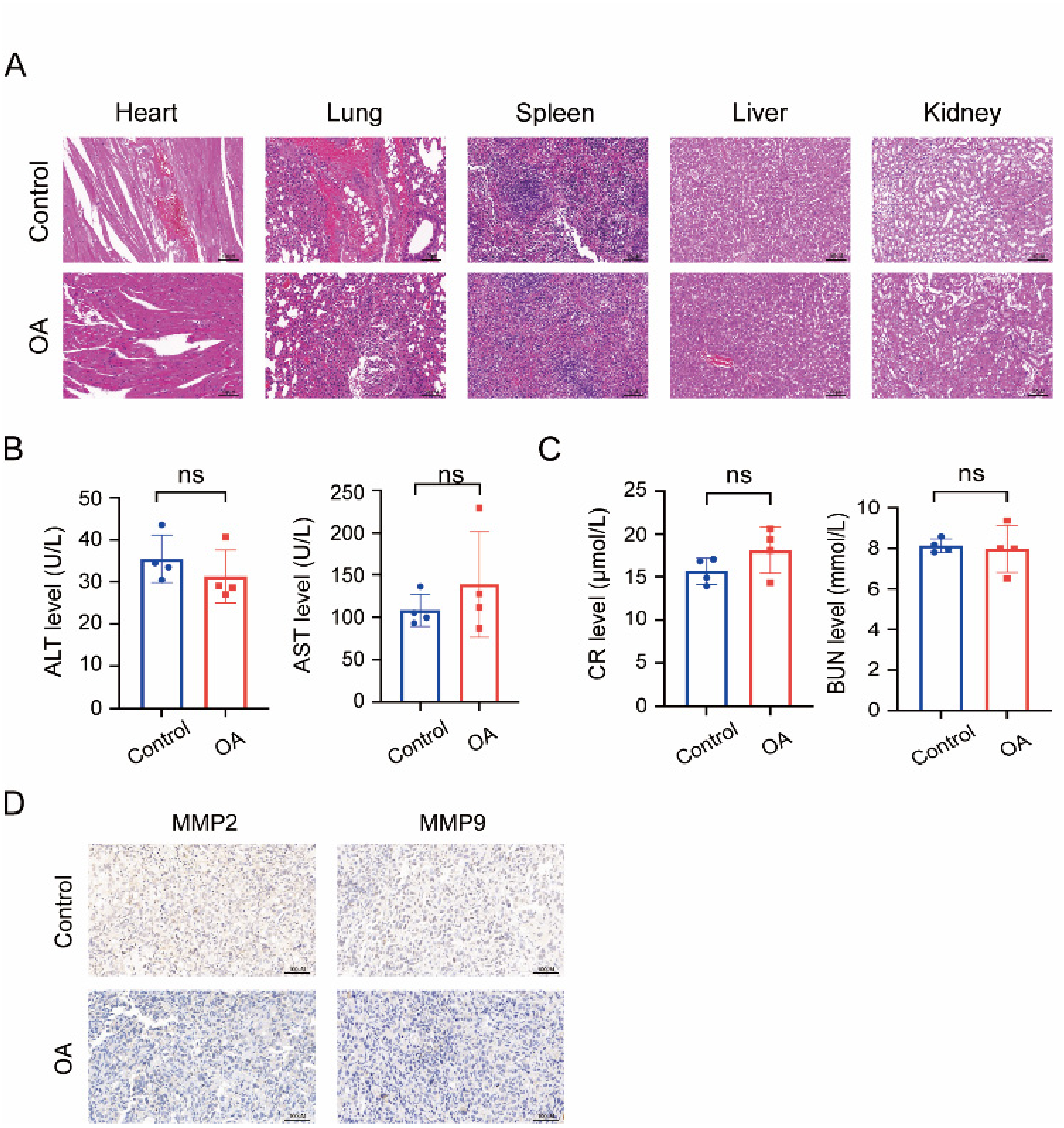

Supplementary Figure S4. H&E-stained histology images of mouse organs and IHC images. **(A)** H&E images of heart, lung, spleen, liver, kidney. **(B)** The levels of ALT and AST. **(C)** The levels of CR and BUN. **(D)** The protein expression of the MMP2 and MMP9 levels in mice tumor tissue were evaluated by IHC.

Supplementary Table S1. MM/GBSA binding free energy components of SRC-Oroxylin A complex.

**Contribution components**	**SRC-Oroxylin A**
$\Delta_{VDWAALS}$	-40.52 ± 1.52
ΔE_{elec}	-21.03 ± 0.67
ΔE_{GB}	26.96 ± 1.76
ΔE_{surf}	-4.93 ± 0.02
ΔG_{gas}	-61.54 ± 1.67
$\Delta G_{solvation}$	22.03 ± 1.76
ΔTotal	-39.52 ± 2.42
