# Supplementary material for: Oroxylin A Directly Targets SRC to Inhibit the PI3K/AKT Signaling Axis in Pancreatic Cancer: An Integrated Bioinformatics and Experimental Study
Source: Biomolecules. 2026 May 5;16(5):685. doi: 10.3390/biom16050685 (PMC13204231; doi:10.3390/biom16050685)

MIA PaCa-2:

BAX Protein：

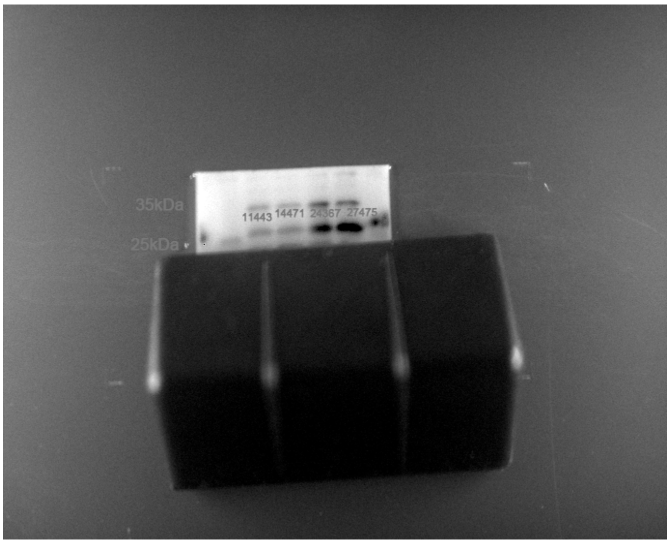


Bcl-2 Protein：

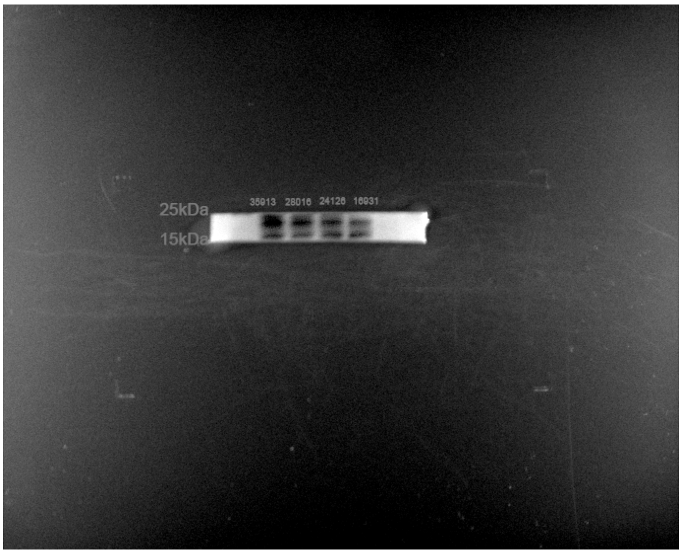


Cleaved caspase 3 Protein

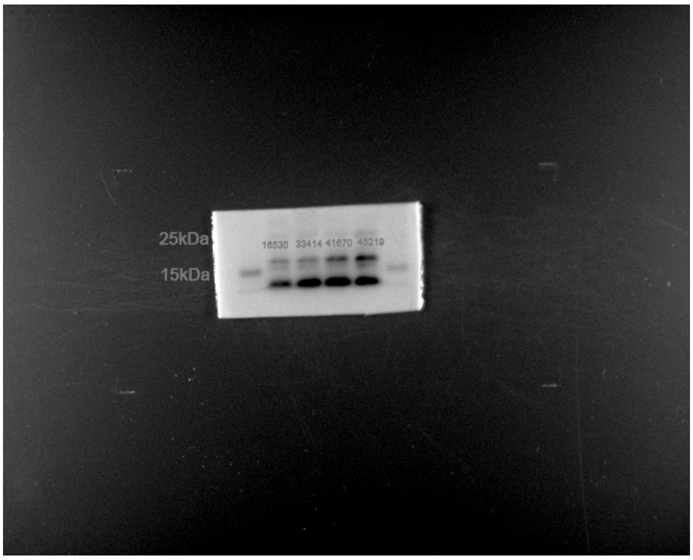


Cleaved PARP

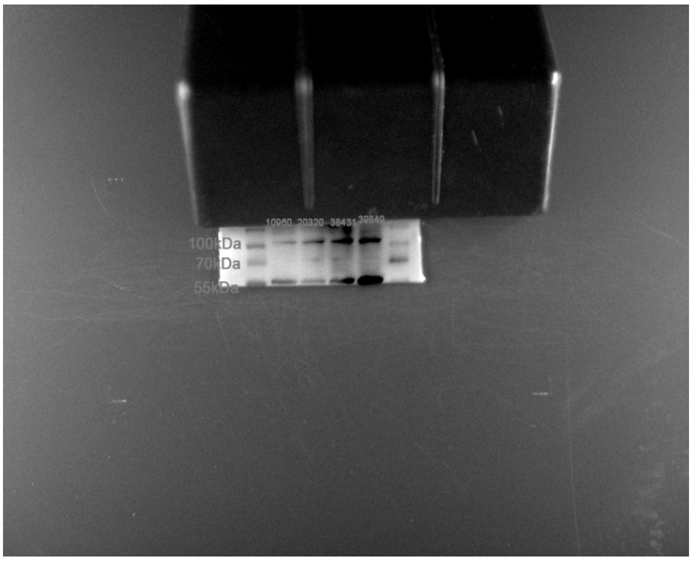


ACTIN：

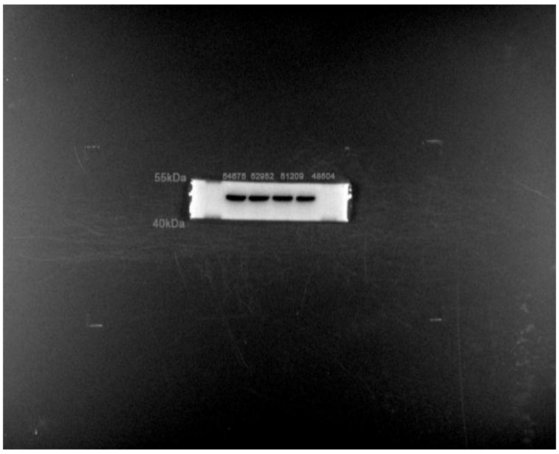

MMP2 Protein：

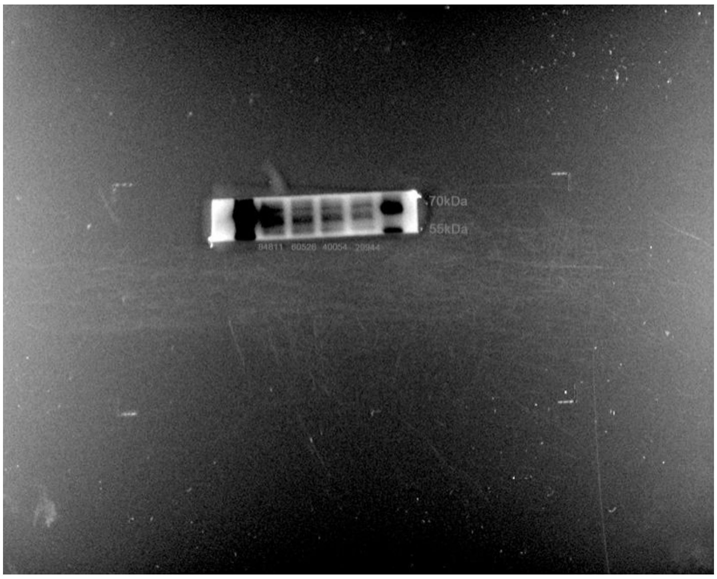


MMP9 Protein：

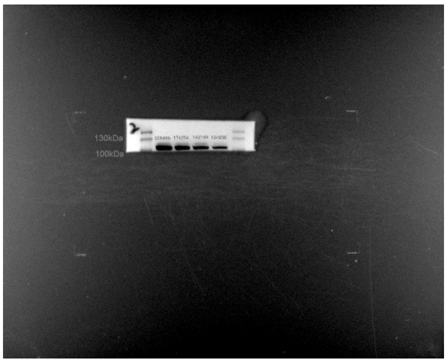


ACTIN Protein：

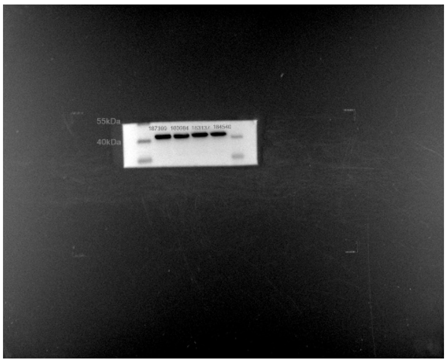


PI3K Protein：

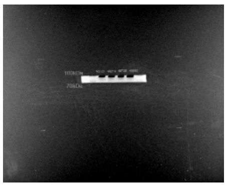

P-PI3K Protein：

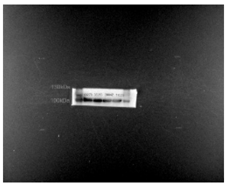

AKT Protein：

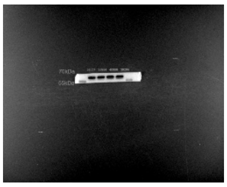

P-AKT Protein：

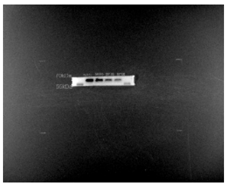

SRC Protein：

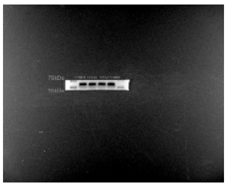

P-SRC Protein：

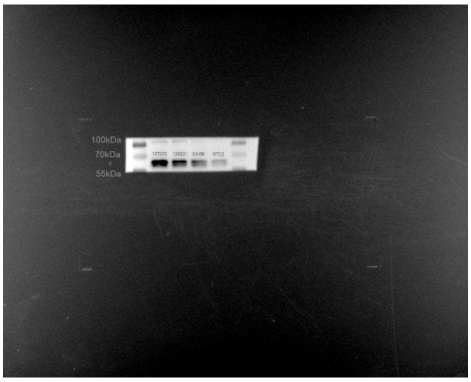


ACTIN Protein：

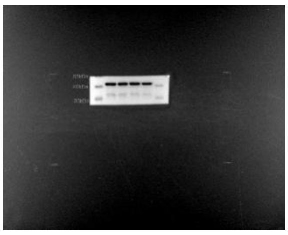

PANC-1：

BAX Protein：

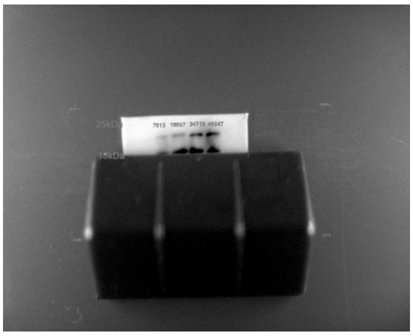


BCL-2 Protein：

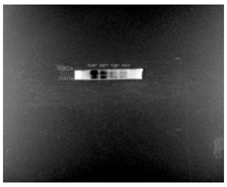

Cleaved caspase 3 Protein：

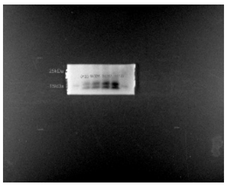

Cleaved PARP Protein：

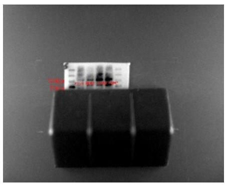

ACTIN Protein：

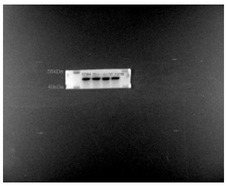

MMP2 Protein：

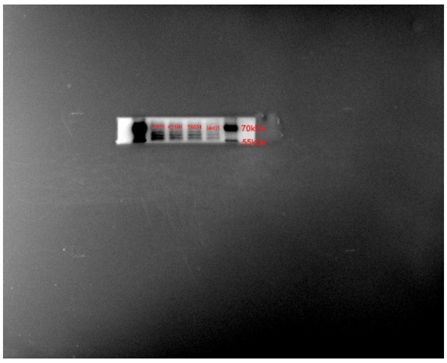

MMP9 Protein：

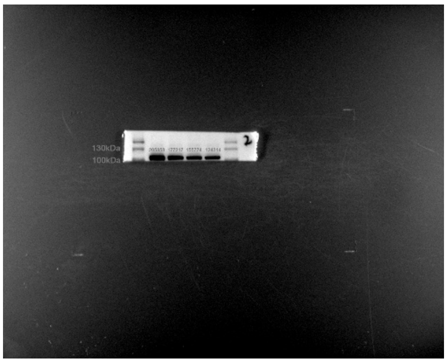


Actin Protein：

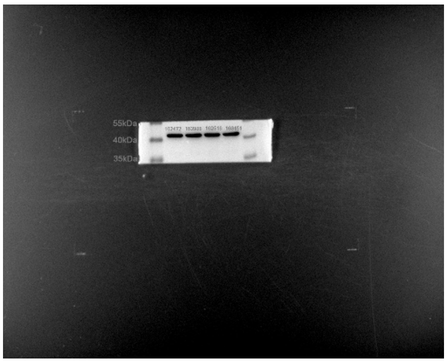


PI3K Protein：

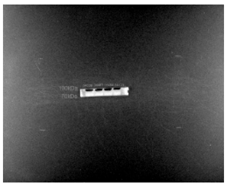

P-PI3K Protein:

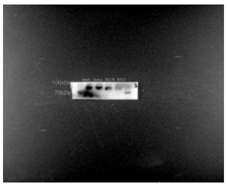

AKT Protein:

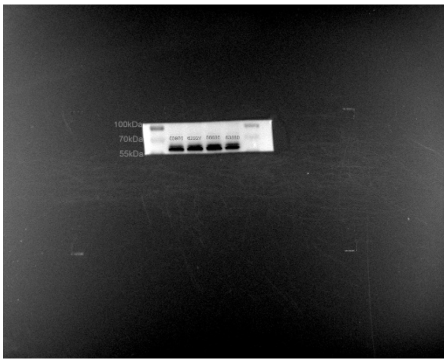


P-AKT Protein:

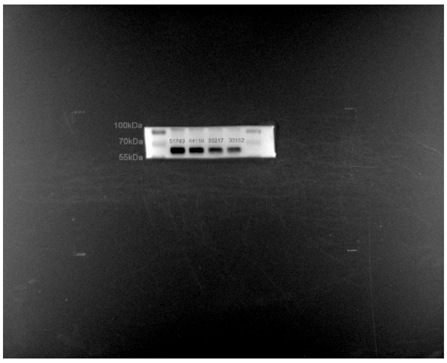


SRC Protein:

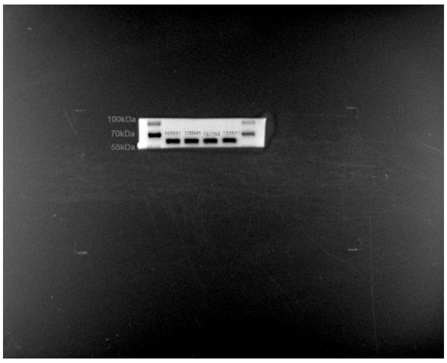


P-SRC Protein:

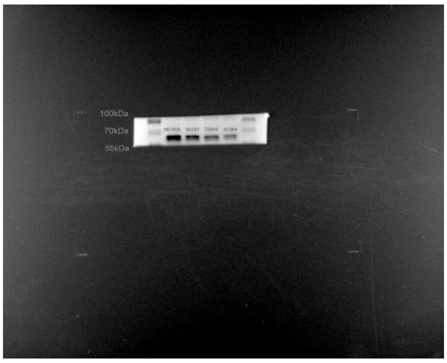


Actin Protein：

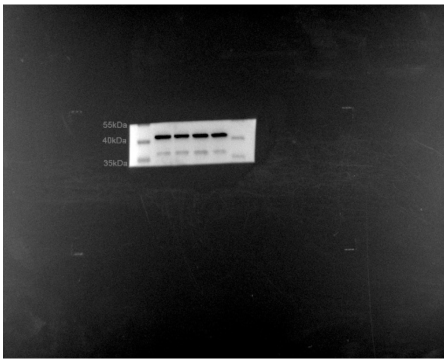


PI3K Protein：

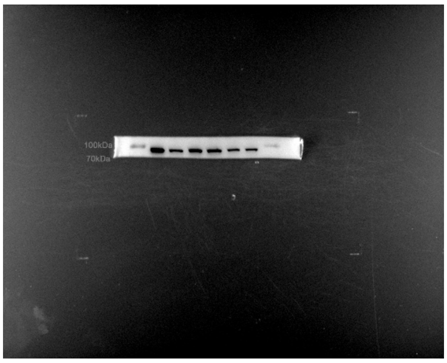


P-PI3K Protein：

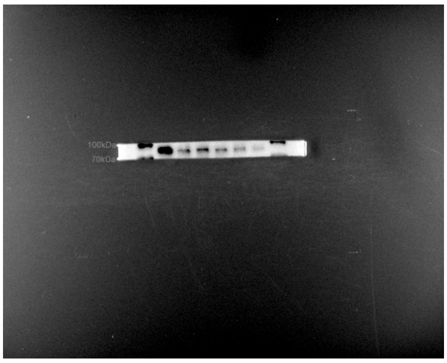

AKT Protein：

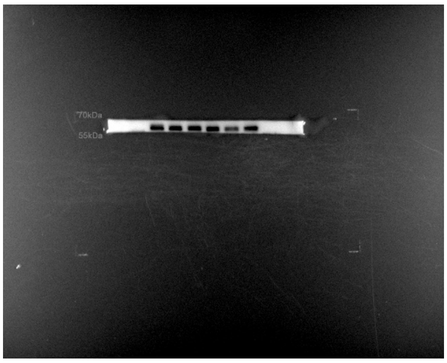


P-AKT Protein：

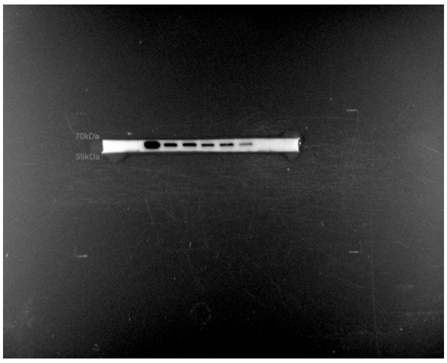


SRC Protein：

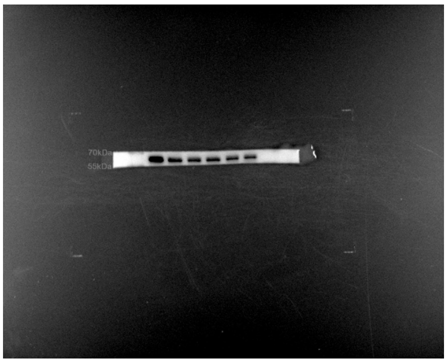

P-SRC Protein：

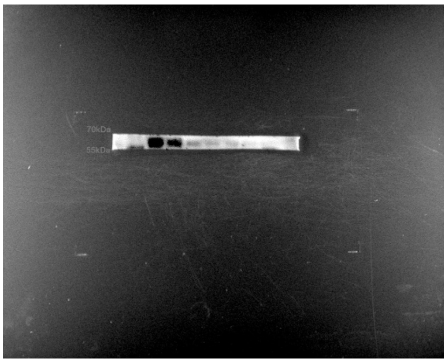

Actin Protein:

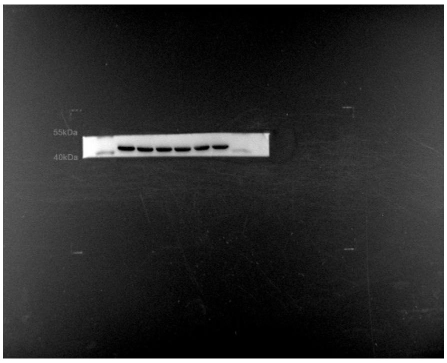

Supplement: Supplementary file 1 [file biomolecules-16-00685-s001.zip › biomolecules-4274030-WB original images revise.pdf]
